# Efficacy of ozone adjuvant therapy in COVID-19 patients: A meta-analysis study

**DOI:** 10.3389/fmed.2022.1037749

**Published:** 2022-11-10

**Authors:** Mehdi Jafari-Oori, Amir Vahedian-azimi, Kobra Ghorbanzadeh, Elham Sepahvand, Manijeh Dehi, Abbas Ebadi, Mortaza Izadi

**Affiliations:** ^1^Atherosclerosis Research Center, Faculty of Nursing, Baqiyatallah University of Medical Sciences, Tehran, Iran; ^2^Trauma Research Center, Nursing Faculty, Baqiyatallah University of Medical Sciences, Tehran, Iran; ^3^Department of Nursing, Khalkhal University of Medical Sciences, Khalkhal, Iran; ^4^Social Determinants of Health Research Center, Poledokhtar School of Nursing, Lorestan University of Medical Sciences, Lorestan, Iran; ^5^Department of Nursing, Maragheh University of Medical Sciences, Maragheh, Iran; ^6^Faculty of Nursing, Behavioral Sciences Research Center, Life Style Institute, Baqiyatallah University of Medical Sciences, Tehran, Iran; ^7^Health Research Center, Life Style Institute, Baqiyatallah University of Medical Sciences, Tehran, Iran

**Keywords:** COVID-19, ozone therapy, standard treatment, systematic review, meta-analysis

## Abstract

**Introduction:**

Using ozone therapy to manage COVID-19 patients has been accompanied by conflicting results in prior studies. Therefore, we aimed to widely assess the effects of ozone as adjuvant therapy in COVID-19 patients.

**Methods:**

PubMed, Scopus, Web of Science, Cochrane, ProQuest, Springer, and Sage journals were searched systematically until April 2022. Mortality rate, ICU admission, hospital-length stay, negative PCR, pulmonary, renal, and hepatic functions, as well as inflammatory and blood systems were pooled to compare the efficacy of ozone as adjacent therapy (OZ) and standard treatment (ST). Analyses were run with the random/fixed models, sub-group analysis, funnel plot, and sensitivity analysis using comprehensive meta-analysis (CMA) software version 2.0.

**Results:**

The results of four randomized clinical trials (RCTs) and four case-control studies with a total of 371 COVID-19 positive patients were analyzed. The OZ group patients had a shorter length of hospital stay (*P* > 0.05), lower ICU admissions (*P* > 0.05), and lower mortality rates (*P* < 0.05) than the ST group cases. After treatment, 41% more COVID-19 patients had negative PCR tests than the ST group (*P* < 0.05). Serum creatinine and urea levels were not modified in either group (*P* > 0.05). Moreover, except for albumin serum levels, which decreased significantly in the OZ group, serum bilirubin, ALT, and AST were not modified in either group (*P* > 0.05). Both arms did not show a decrease in C-reactive protein blood levels (*P* > 0.05), but the OZ group showed a significant modification in LDH serum levels (*P* < 0.05). Unlike the d-dimer and WBC serum levels (*P* > 0.05), platelet levels were increased in the OZ group (*P* < 0.05). No negative side effects were demonstrated in either group.

**Conclusion:**

Ozone therapy was effective significantly on PCR test and LDH serum levels, as well as mortality based on overall estimation. Concerning the length of hospital stay and ICU admissions, although the results were insignificant, their effect sizes were notable clinically. More RCT studies are needed to show the efficacy of ozone therapy on other studied variables.

## Introduction

COVID-19 was initially detected in Wuhan, China, in December 2019. The World Health Organization (WHO) named the pandemic an international public health concern (1–3). COVID-19 has caused more than 570 million cases and 6 million deaths worldwide until July 24, 2022 (4).

Various adjuvant therapies have been used to treat patients with COVID-19, so far, efforts are still continuing to discover the most effective therapy (5, 6). One such adjunctive treatment that has been previously investigated for COVID-19 is ozone therapy (7).

The COVID-19 infection causes an inflammatory response in the lungs, heart, kidneys, and other organs (8). Ozone (O3) has a molecular weight of 48 and a density one and a half times that of oxygen (9). It is a disinfectant gas that boosts the immune system, inhibits viruses from reproducing, and depending on its concentration, has powerful antioxidant effects (10, 11). Ozone reduces inflammation by acting on interleukins, raises ATP in red blood cells, and enhances RBCs’ access to oxygen (12). Additionally, it is an excellent biocidal agent due to its strong oxidizing properties, and its effectiveness has been confirmed in bacteria, fungi, and viruses (13). Ozone can have great potential for improving oxygenation in COVID-19 patients due to its antioxidant, antiviral, and anti-inflammatory characteristics (14). It is administered by various methods, like major and minor hemo-therapy, insufflation, and other methods (15).

The effectiveness of ozone on COVID-19 patients has been studied in several studies (15–17). However, the outcomes of these studies were conflicting, making it difficult for physicians to make a decision on whether to prescribe it or not. Additionally, in several review articles, ozone therapy in COVID-19 patients has been studied; however, these review studies (18–20) except one (21) were not systematic reviews and meta-analyses, and mostly focused on the mechanism of effect. However, in a recent meta-analysis study by Budi et al. (21), the limited outcomes were measured, and the effect of ozone on PCR results, disease severity, pulmonary, hepatic, renal, and hematology profiles have not been addressed. Also, in this study, case reports were also included, while in the present study, only studies that had control and intervention groups were included in the analysis.

Considering the conflicting results in prior studies, the application of ozone in COVID-19 patient’s treatment are remained controversial. Therefore, researchers decided to conduct a systematic review and meta-analysis study to measure the effect of ozone therapy on various factors such as mortality, ICU admission, hospital-length stay, pulmonary, renal, and hepatic functions, as well as inflammatory and blood systems in COVID-19 patients.

## Methods

This systematic review and meta-analysis study was conducted according to the Preferred Reporting Items for Systematic Reviews and Meta-Analyses (PRISMA) statement for systematic reviews and meta-analyses (22). This study’s protocol was registered in PROSPERO with a registration number of CRD42022325049.

### Eligibility criteria

The eligible articles were related to patients with positive RT-PCR (reverse transcriptase-polymerase chain reaction) regardless of the disease severity, included ozone as adjuvant therapy, and published in national or international journals with full text in English or Persian languages from December 1990 to April 2022. Randomized controlled trials (RCTs), clinical trials, cohort studies, and case-control studies with control and intervention groups were included. One group’s studies like case series and case reports were excluded. Furthermore, we excluded review articles, unrelated articles, inaccessible, and duplications.

### Search strategy and study selection

**FIGURE 1:**
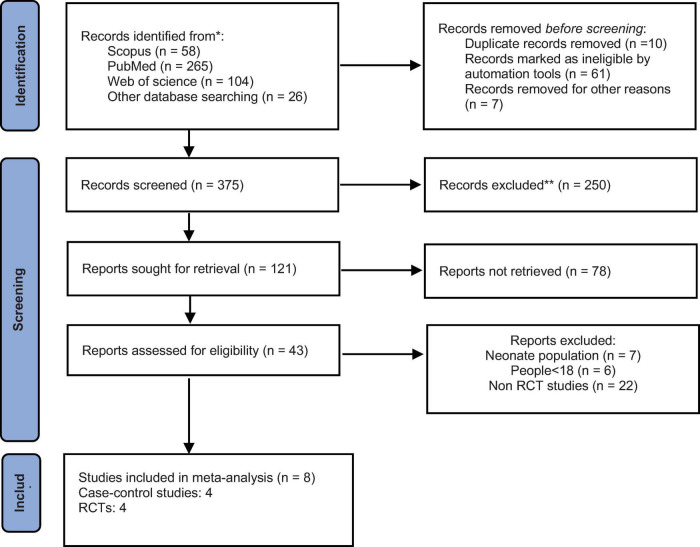
Study selection process.

Initially, MeSH and text terms were identified, and then syntaxes were made according to databases. Two authors (ES and KG) independently searched PubMed, Scopus, the Web of Science, the Cochrane Database of Systematic Reviews, trial registries (ClinicalTrials.gov), and the internal database from December 1990 to April 2022. Google Scholar and Google were also screened as search engines. In addition, reference lists of related articles (backward search) and studies that cited them (forward search) as well as gray literature were reviewed. While conducting a literature search, any controversial ideas were solved by all authors. Selection of studies was fulfilled through conducting three stages of duplicate checking by the reference manager, screening title and abstract to ensure relevancy, and finally screening full text articles to exclude unrelated articles (Figure 1).

### Data extraction

Two review authors (MJ-O and KG), independently extracted the following items: first author’s name, study design, country, sample size, disease severity, types of standard treatments, administration technique of ozone therapy, duration and dose of ozone therapy, adverse effects, and quality score.

### Quality assessment

The revised Cochrane risk-of-bias tool for randomized trials (RoB-2) was used for risk of bias assessment for randomized trials. Bias is judged as high, low, or unclear in terms of selection, performance, attrition, reporting, and other biases (23). We also Newcastle-Ottawa Scale (NOS) to assess case-control studies. Two authors checked the quality of the studies independently (MJ-O and MD), and any discrepancy was resolved by the third author with more capability to review the study (MI).

### Data analysis

To summarize the data, various effect sizes were used, such as risk ratio (RR), risk difference (RD), and mean difference (MD) with 95% confidence intervals. A meta-analysis was run using random effects and fixed effect regarding the level of heterogeneity. The heterogeneity levels were categorized as 0–25, 26–50, 51–75, and 75–100%, indicating low, moderate, and high between-study heterogeneity, respectively (24). In the analyses of homogeneous (I < 50% and *P* > 0.05) and heterogeneous (I > 50% and *P* < 0.05) data, the fixed-effects and random-effects models were used, respectively (25). We were unable to assess publication bias since at least 10 studies are required to assess publication bias (26). A sensitivity analysis using the leave-one-out method was used to analyze the effect of one single research effect on the total pooled estimation. CMA software was used to analyze the data. The *p*-value for statistical significance was set at 0.05. We included both RCTs and case-control studies in a meta-analysis. In many cases, the advantages of integrating both observational studies and RCT in a meta-analysis may outweigh the disadvantages, therefore observational studies shouldn’t be simply excluded (27, 28).

## Results

### Study selection

From databases and manual searches, a total of 1,970 articles were retrieved. Following duplicates check and titles and abstracts screening, 121 articles were remained. After a full-text review, eight studies (2, 6, 8, 17, 29–32) were included in the final analysis. The study selection process is shown in the PRISMA flow chart (Figure 1).

### Study characteristics

Four of the eight studies were RCTs (6, 17, 29, 31), while the remaining four were case-control studies (2, 30, 32, 33). Overall, 371 COVID-19 patients were included, of which 213 and 157 were in the intervention group [Ozone therapy + standard treatment (OZ arm)] and control group [standard treatment (ST)] as a standard, respectively. The lowest (*n* = 18) and highest (*n* = 92) sample sizes were related to studies by Hernandez et al. (34), respectively. Articles were from Italy (29, 31, 32), Spain (8, 30), Turkey (2, 17), and India (6). Patients eligible for this study were aged 18 or older, hospitalized with positive PCR tests, ranked mild to severe on the National Early Warning Score (NEWS score 8), and were spontaneously breathing ambient air, using a venturi mask, high flow nasal cannula, or continuous positive airway pressure (CPAP). All samples were scored 2 or 3 based on the Italian Society of Emergency and Urgent Medicine’s (SIMEU) COVID-19 classification, and all had severe pneumonia. Ozone was administrated by different techniques including major autohemotherapy (MAH) (2, 6, 8, 29, 31), rectal insufflation with minor autohemotherapy (6), rectal insufflation (30), and ozone nebulization (17). Corticosteroids, antivirals (lopinavir, ritonavir, and remdesivir), antibiotics (like azithromycin), and vitamin supplements (vitamin E, vitamin C, vitamin D, and zinc) comprise up the standard treatment regimens (2, 6, 17, 29–32, 34). Table 1 shows the detailed characteristics of the included studies.

**TABLE 1:** Basic characteristics of included studies.

Author	Study design	Country	Sample	Disease severity	Types of Baseline treatment	Type of ozone administration	Duration and dose of ozone therapy	O3 adverse effect	Outcome type
Çolak et al. (2)	Case-control study	Turkey	55 patients (OZ + ST: 37, ST: 18)	Patients with respiratory system complaints.	Hydroxychloroquine, enoxaparin, favipiravir, and antibiotics	Ozone major autohemotherapy (MAH)	Seven sessions (one session per day), in a volume of 100 mL and a concentration of 30 μg/mL	No	Mortality, ICU admission
Shah et al. (7)	RCT	India	60 patients (OZ + ST: 30, ST: 30)	Mild to moderate score based on NEWS score	Indian Council of Medical Research (ICMR) protocol	Ozonized rectal insufflation and [minor auto haemotherapy (MiAHT)]	10 days; 40 A^∧^μg/ml ozone in the dose of about 150 ml twice daily as a rectal insufflation, and 2–3 ml venous blood along with 5 ml ozone at 25 A^∧^μg/ml	No	Clinical features, NEWS score (RT-PCR), inflammatory markers, the requirement of advanced care, and metabolic profiles
Fernández-Cuadros et al. (29)	Case-control study	Spain	28 patients (OZ + ST: 14, ST: 14)	Severe COVID-19	Antivirals, corticosteroids, antibiotics, anticoagulants, and anti-IL-6	Rectal ozone, 8 sessions (1 session/day)	for 5 to 10 days, insufflation of a volume of 150 mL at a concentration of 35 μg/mL	No	Clinical, biochemical, radiological Taylor’s scale, hospitalization length of stay, ad mortality
Aramio et al. (28)	RCT	Italy	28 patients (OZ + ST: 14, ST: 14)	Respiratory supported with venturi mask (VMK) or HFNC, or CPAP	Antivirals, corticosteroids, and antibiotics	MAH daily double treatment until 7 days	Seven days, A total of 15 × 103 mcg of ozone was the daily	No	Tracheal intubation, mortality, hematological parameters
Dengiz et al. (16)	RCT	Turkey	30 patients (OZ + ST: 15, ST: 15)	PCR positive patients admitted to the emergency department	Antivirals, corticosteroids, and antibiotics.	Ozone inhalation	Five days, three sessions applied for 10 mins at intervals of 5 mins daily. Each session; 0.2 ppm ozone	No	Clinical and biochemical tests
Sozio et al. (30)	RCT	Italy	92 patients (OZ + ST: 48, ST: 44)	Mild to moderate pneumonia based on SIMEU clinical phenotype (2 or 3)	Antivirals, corticosteroids, and antibiotics	MAH	For 3 days, 200 mL of a gas mixture composed of 96% of Oxygen and 4% of ozone with a therapeutic O3 range of 40 μg/mL of gas per mL of blood	No	Hospital stay; improved chest imaging, oxygen therapy, CPAP, tracheal intubation, and inflammatory response
Tascini et al. (31)	Case-control study	Italy	60 patients (OZ + ST: 30, ST: 30)	Moderate pneumonia based on SIMEU clinical phenotype (2 or 3)	Antivirals, corticosteroids, and antibiotics	MAH	Three days, 200 ml freshly prepared ozonized saline intravenously over 1 h once a day for 8 days along with standard medical treatment	No	Clinical and biochemical tests
Hernandez et al. (33)	Case-control study	Spain	18 patients (OZ + ST: 9, ST: 9)	Severe COVID-19 pneumonia	Antivirals, corticosteroids, and antibiotics	MAH	Five days, 200 mL blood of oxygen-ozone mixture with a 40 μg/mL ozone concentration	No	Time from hospital admission to clinical improvement

### Studies’ risk of bias

The study’s risk of bias result indicated that three RCTs were high-quality (6, 17, 31), whereas one seemed to has a little risk of bias (29) regarding the Cochrane’s Risk of Bias 2 (RoB2) assessment (Supplementary Table 1). Furthermore, based on the NOS, three-quarters of case-control studies were high-quality (30, 32, 34), and the other one was medium-quality (2) (Supplementary Table 2).

### Comparison of the effect of ozone therapy and standard treatment for the management of COVID-19 patients

The effects of OZ and ST were assessed and compared in terms of hospital stay, ICU admission, mortality rate, renal and hepatic profiles, inflammation markers and hematology profile, Reverse transcription-polymerase chain reaction (RT-PCR), and safety parameters in COVID-19 patients, which are reported in the following.

### Length of hospital stay

**FIGURE 2:**
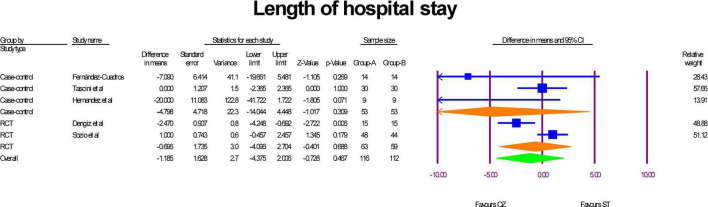
A forest plot of hospital stay lengths based on study type, comparing OZ and ST groups (Group A = OZ, Group B = ST).

The length of hospital stay was reported in two RCTs (17, 31) and three case-control studies (30, 32, 34). The sub-group analysis indicated that patients who were treated with Ozone as an adjuvant therapy were hospitalized less than ST-treated patients both in RCT studies (MD = –0.69 day, *P* = 0.46, I^2^ = 0.88, random-effects) and case-control (MD = –4.79 days, *P* = 0.30, I^2^ = 0.53, random-effects) studies; however, the results were statistically insignificant. Additionally, the overall analysis of both study types in a single mate-analysis indicated that the Ozone treated patients were hospitalized insignificantly about 1 day less than the standard treated patients (MD = –1.18 day, *P* = 0.46, I^2^ = 0.69, random-effects) (Figure 2). Statistical insignificance and heterogeneity were maintained after the run of the leave-one-out sensitivity analysis. Egger’s regression intercept test was –1.72 (*p* = 0.120), indicating no publication bias (Supplementary Figure 1).

### ICU admission

**FIGURE 3:**
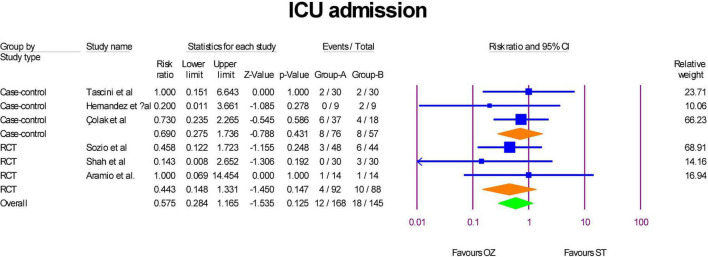
A forest plot of ICU admission based on study type in the OZ group compared to the ST group (Group A = OZ, Group B = ST).

ICU admission were reported in the three RCTs (6, 29, 31) and three case-control (2, 32, 34) studies. As Figure 3 show, although RR of ICU admission in RCTs (RR = 0.44, *P* = 0.14, I^2^ = 0, fixed-effect) and case-control (RR = 0.69, *P* = 0.43, I^2^ = 0, fixed-effect) studies were less in the Ozonized patients than the ST group, but the differences were not statistically significant. Similarly, the overall RR of ICU admission was in favor of OZ group, but the result was insignificant (RR = 0.57, *P* = 0.123, I^2^ = 0, fixed-effect). The significance of the results was not established by the sensitivity analysis. There was no evidence of publication bias in the funnel plot (Egger’s regression intercept = –0.73, *p* = 0.371; Supplementary Figure 2).

### Mortality

**FIGURE 4:**
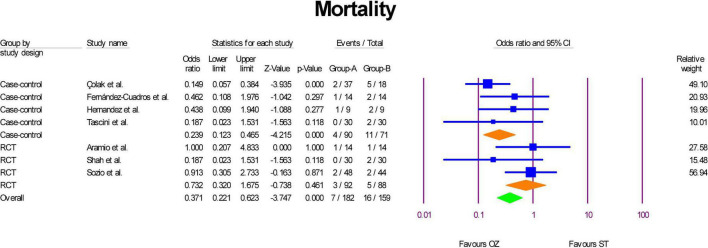
Forest plot of mortality rate in the OZ arm compared with the ST arm.

Mortality was measured in three RCTs (6, 29, 31) and four case-control studies (2, 30, 32, 34). As indicated in Figure 4, the pooled mortality of case-control studies revealed that the patients in the Ozone group had significantly about 0.76% less mortality than the patients in the ST group (OR = 0.23, *P* = 0.000, I^2^ = 0, fixed-effect). However, according to the pooled estimation of RCTs, Ozone therapy was not associated significantly with odds of mortality than the standard therapy (OR = 0.73, *P* = 0.4610, I^2^ = 0, fixed-effect). When leave-one-out sensitivity analyses were carried out, neither heterogeneity nor statistical significance were affected. Considering the overall pooled estimation, standard treatment patients had high odds of mortality than the Ozone therapy patients (OR = 0.37, *P* = 0.000, I^2^ = 30.48, fixed-effect). The funnel plot and Egger’s test showed no publication bias (Egger’s regression intercept = 0.35, *p* = 0.851; Supplementary Figure 3).

### RT-PCR

**FIGURE 5:**
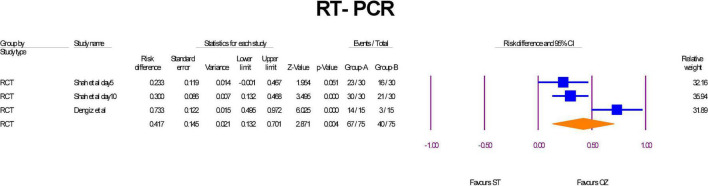
Forest plot of negative RT-PCR in the ST arm compared with the OZ arm.

Two RCTs examined RT-PCR (6, 17). RT-PCR was measured two times at day 5 and day 10 after finishing of Ozone therapy in the study of Shah et al. (6). The results of those both times results were included. The number of COVID-19 patients who revealed negative PCR in the OZ arm was significantly higher than the patients in the ST group (RD = –0.41, *P* = 0.004, I2 = 81.44, random-effects) (Figure 5). The leave-one-out sensitivity analysis displayed that the statistical significance was canceled if Shah et al., Day 10 (6) was removed. As the Supplementary Figure 4 shows, there was no publication bias (Egger’s regression intercept = 5.61, *p* = 0.671).

### Renal profile

**FIGURE 6:**
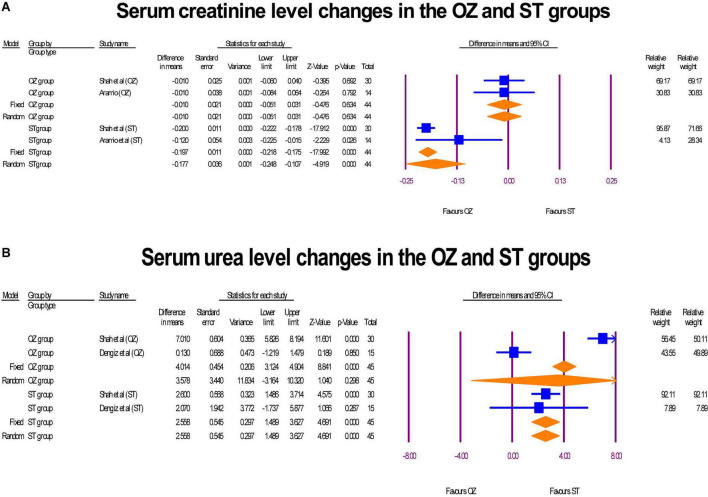
The forest plots of serum creatinine **(A)** and urea level **(B)** changes in the both arms.

Two RCTs reported serum creatinine (mg/dL) and Urea (mg/dL) level changes before and after of intervention in the both OZ and ST arms (6, 29). There was an insignificant reduction in serum creatinine levels among patients receiving ozone therapy (MD = –0.01 mg/dL, *P* = 0.634, I2 = 0, fixed-effect), whereas for the ST group, this change was significant, suggesting that serum creatinine levels declined significantly after the intervention (MD = –0.17 mg/dL, *P* = 0.000, I2 = 52, random-effects) (Figure 6A). Additionally, as shown in Figure 6B, in the OZ arm, the pooled MD of serum level of BUN increased non-significantly (MD = 3.58 mg/dL, *P* = 0.298, I2 = 98, random-effects), indicating that ozone therapy resulted in elevated serum urea levels in patients. A significant increase in serum urea levels was also observed after treatment in the ST group (MD = 2.53 mg/dL, *P* = 0.000, I2 = 000, fixed-effect).

### Hepatic profile

**FIGURE 7:**
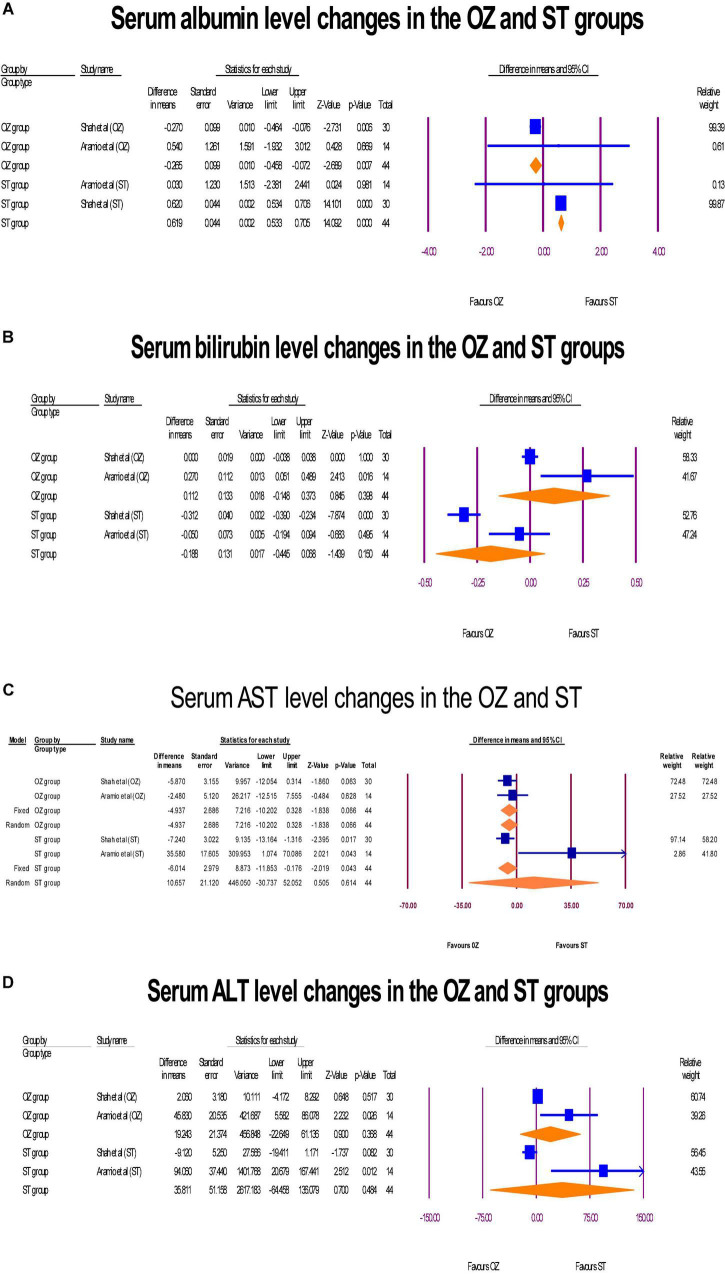
The forest plots of hepatic profile changes **(A–D)** before and after the interventions in the both groups.

The changes of hepatic markers levels including albumin (g/dL), bilirubin (mg/dL), AST [international units per liter (IU/L)], and ALT (IU/L) were measured in two RCTs (6, 29). Regarding to the albumin, a significant change was exhibited in the serum albumin levels. However, the albumin level decreased in the OZ group (MD = –0.26 g/dL, *P* = 0.007, I2 = 0, fixed-effect), and increased in the ST group (MD = 0.61 g/dL, *P* = 0.007, I2 = 0, fixed-effect) (Figure 7A). Further, neither in the OZ (MD = 0.11 mg/dL, *P* = 0.150, I2 = 82.31, random-effects) nor in the ST (MD = 0.18 mg/dL, *P* = 0.398, I2 = 89.31, random-effects) groups, the interventions were not associated with total bilirubin levels changes (Figure 7B). Likewise, the serum AST level was increased non-significantly in the OZ group (MD = –4.93 IU/L, *P* = 0.066, I2 = 0, fixed-effect) and but decreased in the ST group (MD = 10.70 IU/L, *P* = 0.614, I2 = 82.36, random-effects) (Figure 7C). Moreover, a non-significant decrease in ALT levels was observed in the both the OZ (MD = 19.24 IU/L, *P* = 0.368, I2 = 77.46, random-effects) and ST (MD = 35.81 IU/L, *P* = 0.484, I2 = 86.57, random-effects) groups (Figure 7D).

### Inflammation markers

**FIGURE 8:**
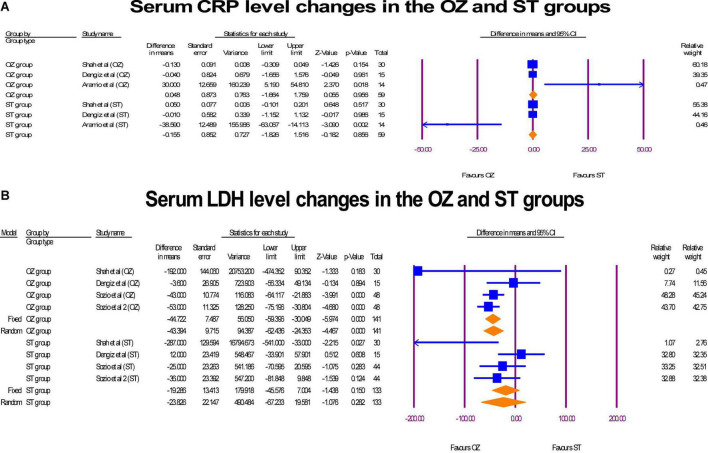
The forest plots of inflammatory markers changes before and after of interventions in the both arms.

A pre- and post-intervention analyses of inflammation markers including CRP (mg/L) and LDH [units per liter (U/L)] were performed in three RCTs (6, 17, 29). In the Ozone-treated arm, a decrease in the serum CRP levels was observed (MD = 0.04 mg/L, *P* = 0.966, I2 = 64.76, random-effects), whereas, a reverse change was detected in the ST group (MD = –0.15 mg/L, *P* = 0.866, I2 = 79.12, random-effects) (Figure 8A). The sensitivity analysis showed that the study of Aramio et al. (29), had considerable effect on the polled CRP serum level changes. Both arms reached zero heterogeneity after removing it, but statistically significant levels were not changed. Further, a significant modification in the LDH serum levels was seen in the OZ group (MD = –44.72 U/L, *P* = 0.000, I2 = 23.87, fixed-effect), but the ST arm had a lower but non-significant modification in LDH level (MD = –23.85 U/L, *P* = 0.282, I2 = 54.70, X2 = random-effects) (Figure 8B). If Shah et al. (6) was omitted, heterogeneity level (I2 index) was decreased from 54.70 to 13.39 in ST arm. In funnel plots of CRP (Supplementary Figure 4) and LDH (Supplementary Figure 5), no publication bias was observed (*p* > 0.05).

### Hematology profiles

**FIGURE 9:**
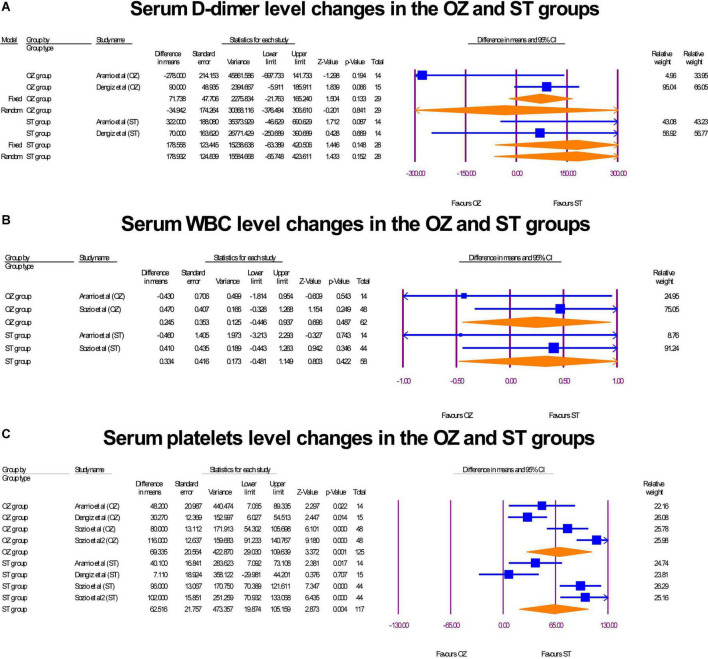
The forest plots of hematology profile changes before and after interventions in the both arms.

Serum D-dimer (ng/mL) was assessed in two RCTs (17, 29). Also, WBC (10^9^/L) and platelets (10^9^/L) serum level were reported in two RCTs (29, 31) and three RCTs (17, 29, 31), respectively. In the OZ group, D-dimer levels decreased, but the change value was not statistically significant (MD = –34.94 ng/mL, *P* = 0.0201, I^2^ = 64.36, random-effects). Likewise, an insignificant increase in D-dimer levels was shown in the ST-treated arm (MD = 178.55 ng/mL, *P* = 0.148, I2 = 2.13, fixed-effect) (Figure 9A). The WBC serum levels both in the pre and post intervention in both groups were in the normal range (4.5–11.0 × 10^9^/L). Neither the Ozone therapy (MD = 0.24 × 10^9^/L, *P* = 0.487, I2 = 2.13, fixed-effect) nor standard medications (MD = 0.33 × 10^9^/L, *P* = 0.422, I2 = 0, X2 = 0.45, fixed-effect) had a significant effect on the serum WBC level (Figure 9B). Finally, Both the OZ (MD = 69.33 × 10^3^ per microliter of blood, *P* = 0.001, I2 = 81.44, random-effects) and ST (MD = 62.51 × 10^3^ per microliter of blood, *P* < 0.05, I2 = 86.50, random-effects) groups had a significant increase in platelet count, after the interventions (Figure 9C). No publication biases were observed in platelet reported studies (Egger’s regression intercept = –2.45, *p* = 0.81; Supplementary Figure 4). The degree of heterogeneity did not change when a leave-one-out sensitivity analysis was performed.

### Comparison of safety parameters between the groups

In the study by Aramio et al. (29), no adverse effect was noticed in the OZ arm. However, 30% of the control group revealed gastrointestinal disorders, such as diarrhea. Similarly, Fr et al. (30) reported that after eight sessions of rectal ozone therapy, clinical, biochemical, and radiological improvement were witnessed. No side effects were reported except a feeling of bloating, which diminished spontaneously. Likewise, other studies reported no safety issues in the OZ groups.

## Discussion

The therapeutic efficacy of OZ in combination with ST was evaluated in COVID-19 patients in this study. Despite the fact that our meta-analysis study demonstrated that ozone adjuvant therapy improved clinical variables and laboratory biomarkers in COVID-19 patients, except for mortality rates, PCR tests, and serum levels of LDH, its effects were insignificant. From a clinical perspective, the estimated effect sizes of the variables can be notable, regardless of the significance level.

Ozone treatment was found to be more beneficial than usual care in COVID-19 patients with severe respiratory symptoms (35). According to Franzini’s research, O2 saturation increased from 85 to 95% after an 8.6-day ozone treatment (13). Araimo also discovered that the demand for ventilator support was moderately reduced in the ozone group (reduced need for CPAP, high-flow nasal cannula, or venturi masks) (29). The same result was observed in Schwartz’s study, in which supplemental oxygen usage dropped from 68 to 24% in the OZ arm (36). The amelioration of bilateral radiographic pneumonia based on Taylor’s radiologic scale could be one cause for enhancing O2 saturation and minimizing the demand for O2 supply in the OZ arm (35, 36).

Other studies have found that the OZ arm’s hospital stay is shorter than the ST group’s, which supports our findings (16, 33, 36).

Similarly, previous studies have found that COVID-19 patients who received ozone therapy had a reduced mortality rate than ST patients (8, 32, 36). According to our findings, ozone’s potential antiviral activity can aid in the early reduction and clearance of COVID-19, resulting in less virus infiltration and harm to organs (37). Additionally, some other one-group trials’ results supported the statistically significant effect of OZ therapy in COVID-19 patients. For example, Schwartz et al. reported that no one who received treatment with ST + OZ passed away temperature (36). Alhmadi Hekmat in his study (38) demonstrated no significant change in mortality after OZ therapy. An increase in mortality could result from the administration of the medication at a late stage of the disease or when multi-organ dysfunction is present.

In terms of renal indicators, there was no significant change in the OZ arm, but a minor significant improvement in the ST group. In this meta-analysis, the effect of ozone therapy on creatinine level was investigated in two primary studies (6, 29). In Shah et al.’s study, serum creatinine level at baseline was 0.78 ± 0.27, which declined to 0.77 ± 0.17 after ozone therapy (6). Similarly, Aramio et al. indicated that the creatinine level was 0.84 ± 0.24 and 0.83 ± 0.18 at baseline and 7 days after starting ozone therapy, respectively (29). In the control group, this change was from 1 ± 0.14 to 0.80 ± 0.13 in the Shah et al.’s study (6), and from 0.97 ± 0.28 to 0.85 ± 0.22 in the Aramio et al.’s study (29). In both studies, creatinine levels were at a normal range in both groups before and after the interventions. Likewise, a study showed insignificant serum creatinine level change after OZ therapy (16).

Regarding BUN, of three studies (6, 17, 35), only in one study (35), BUN serum level was over the upper limit, but it modified slightly in favor of ozone. As a result of these findings, ozone therapy may have a beneficial effect on kidney function.

On the hepatic profile, serum albumin level was slightly amended in the OZ group but significantly increased abnormally over the upper limit in the ST group, reflecting more hepatic damage. Also, the total bilirubin level was in the normal range before and after the interventions in both groups. In light of these findings, it is currently difficult to determine what effect ozone therapy can have on the total bilirubin level. Likewise, the AST level was in the normal range both pre-and post-intervention in the OZ arm but did not change significantly after ozone therapy, whereas it had increased to an abnormal upper level in the ST group. Similarly, Although the amount of ALT was insignificantly increased abnormally in both groups; however, this rate of change was higher in the ST group than in the OZ arm. Hepatic profiles were assessed only in two RCT studies (6, 29); more research is needed to get a more conclusive result.

In terms of inflammatory indicators, the level of CRP and LDH in the ozone group decreased dramatically. In terms of magnitude, this amount of reduction was more than the ST group. The rate of LDH reduction was not statistically significant in the ST group. Other research has shown that ozone can modify interferons, cytokines, and inflammation biomarkers (39, 40). In other one group experimental studies, contrary to findings of Franzini et al. (16), Sharma et al. (14), administered ozone adjuvant therapy on 10 patients intravenously over 1 h once a day for 8 days, the results showed that the change in LDH was borderline statistically not significant (*p* = 0.058).

In the case of the hematological profile, the amount of WBC was in the normal range in both groups before and after the intervention, and the interventions had no significant influence on it. However, platelet levels increased significantly and equally in both intervention groups. Bocchi claims that ozone promotes the differentiation of white blood cells and platelets in addition to activating stem cells (41).

In terms of safety parameters, this review found no evidence of a harmful effect from ozone, which is consistent with a manuscript that found just 0.7 recorded adverse reactions per 100,000 treatments (42). Also, ozone did not negatively affect any extra pulmonary organs, such as the hepatic, kidneys, lipid profile, or blood cell profile (6).

### Limitation

This meta-analysis included a small number of studies, which was just eight studies. Also, ozone was administered by autohemotherapy, rectal insufflation, and inhalation, which may lead to different levels of effect. Further studies are needed to provide estimated effect measures based on administration type. Similarly, only non-mechanically ventilated patients were in the included RCT studies. Based on the positive therapeutic effect of ozone in COVID-19 patients, it appears that the effect of ozone in critically sick patients, who are intubated, should be examined as well. Because the goal of our analysis was to compare the therapeutic effects of ozone to standard treatment, we only included RCTs with OZ and ST arms and excluded studies with alternative designs such as case reports, case series, and single-group semi-experimental studies. We were unable to undertake subgroup analysis or meta-regression to control and reduce heterogeneity between findings due to the limited number of RCT trials. Despite the mentioned limitations, this is presently the first comprehensive meta-analysis study that has been able to examine a wide range of clinical and biochemical effects of ozone, as well as identify knowledge gaps that should be addressed by future research.

## Conclusion

Although our study showed that, in most cases, ozone adjuvant therapy was insignificant in COVID-19 patients, the estimated effect sizes were notable. Based on the safety parameters of ozone adjuvant therapy, its administration in COVID-19 patients may result in positive results. However, more research is needed to understand the real effects of ozone adjuvant therapy on laboratory and clinical outcomes.

## Author contributions

MJ-O, KG, ES, MD, AE, and MI: idea. MJ-O and MI: literature search. MJ-O, KG, and ES: analysis. MJ-O and AV-A: first draft of the manuscript. MJ-O, AV-A, AE, and MD: final draft of the manuscript. MJ-O, AE, and MI: supervision. All authors contributed to the article and approved the submitted version.
