# Supplementary material for: Efficacy of ozone adjuvant therapy in COVID-19 patients: A meta-analysis study
Source: Front Med (Lausanne). 2022 Nov 10;9:1037749. doi: 10.3389/fmed.2022.1037749 (PMC9685165; doi:10.3389/fmed.2022.1037749)
Supplement: Supplementary file 1 [file Data_Sheet_1.pdf]

Table S1. Quality assessment of ROB.2 for RCTs studies

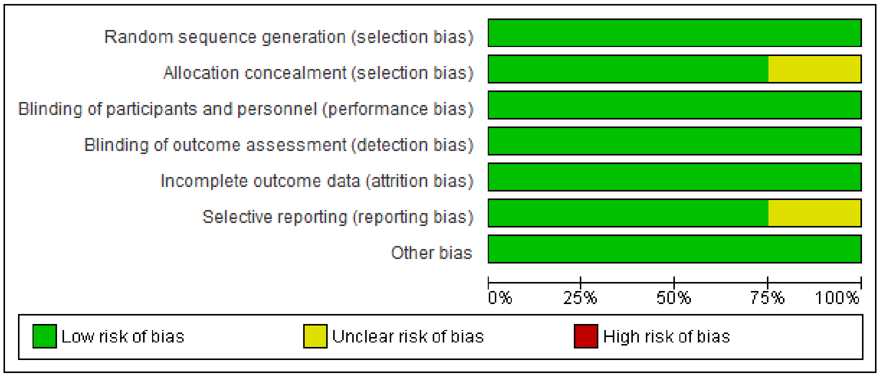

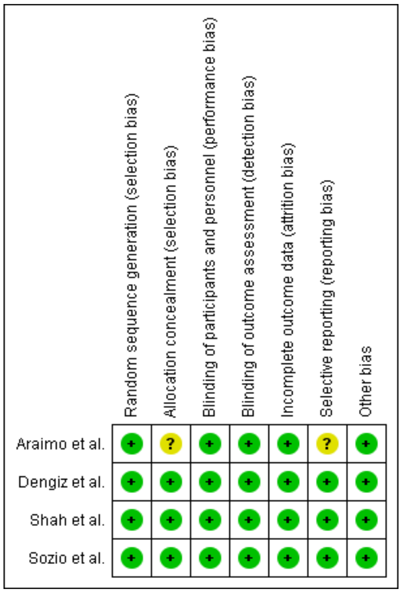

Table S2. NOS for Case-Control Studies

Studies	Selection				Comparability	Exposure			Total Score	RESULT
	**Is the case definition adequate?**	**Representativeness of the cases**	**Selection of Controls**	**Definition of Controls**	**Comparability**	**Ascertainment of exposure**	**Same method of ascertainment for cases and controls**	**Non-Response rate**		
Tascini *et al.* [18]	✓	✓	✓	-	✓	✓	✓	✓	**7**	High Quality
Fernández-Cuadros *et al.*[19]	✓	✓	✓	-	✓	✓	✓	✓	7	High Quality
Hernández *et al.* [20]	✓	✓	✓	-	✓	✓	✓	✓	7	High Quality
Çolak *et al.* [21]	✓	✓	✓	-	✓	✓	✓	✓	6	Medium Quality

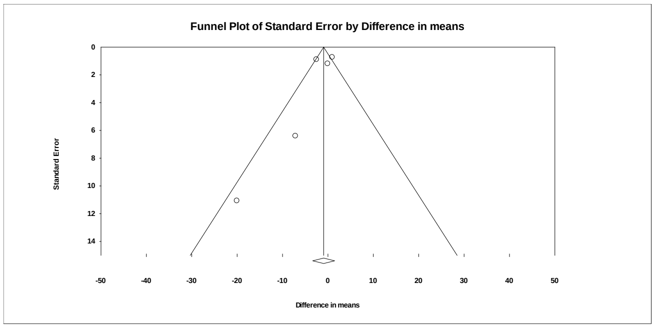

Figure S1: Funnel plot of publication bias on length of hospital stay

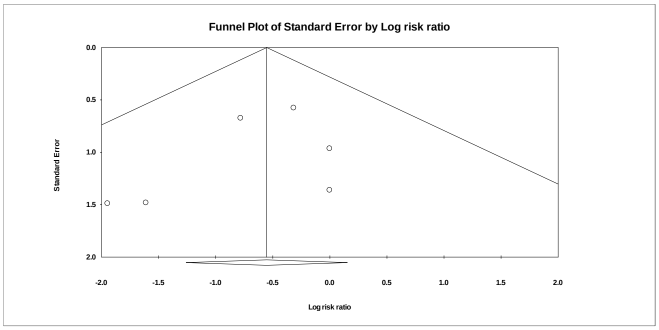

Figure S2: Funnel plot of publication bias on ICU admission

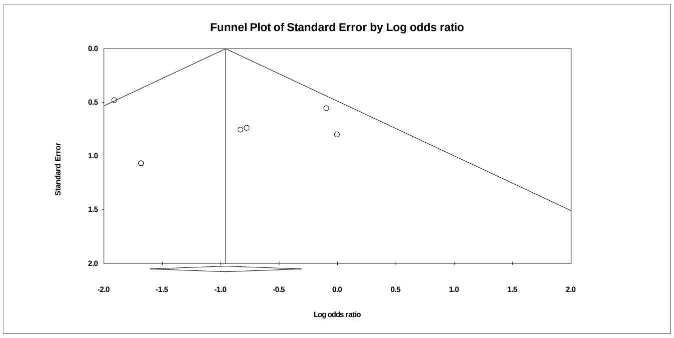

Figure S3: Funnel plot of publication bias on mortality

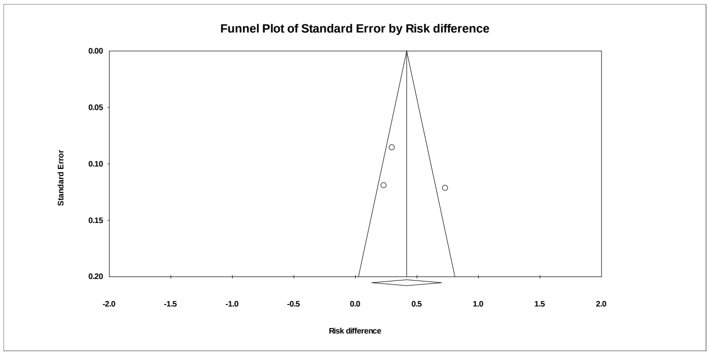

Figure S4: Funnel plot of publication bias on RT- PCR

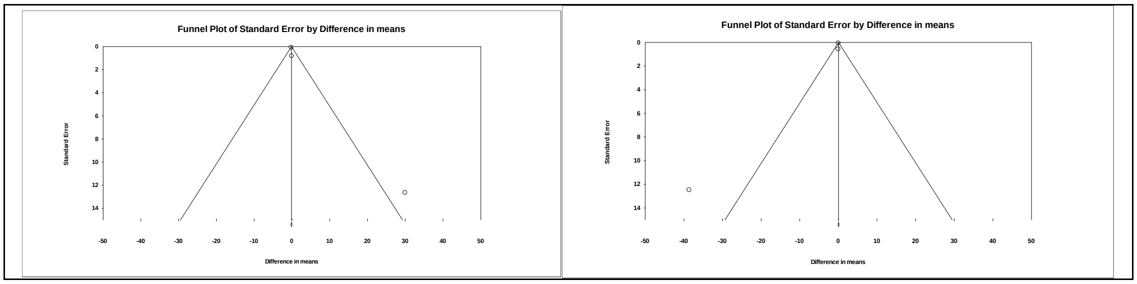

Figure S5: Funnel plot of publication bias on CRP for OZ (left) and ST (right) groups

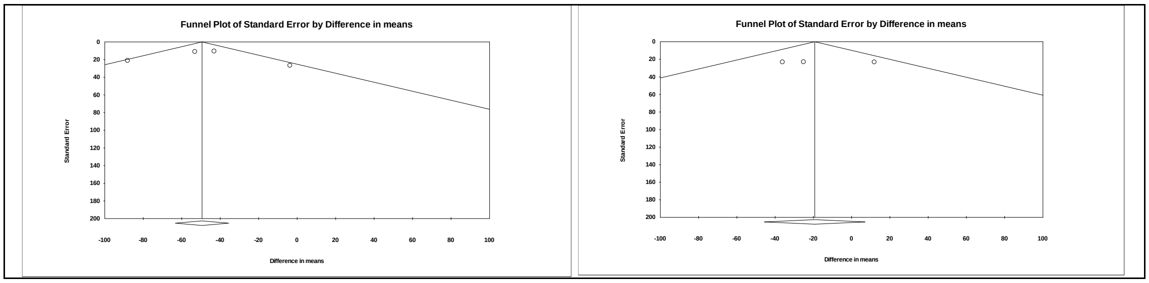

Figure S6: Funnel plot of publication bias on LDH for OZ (left) and ST (right) groups

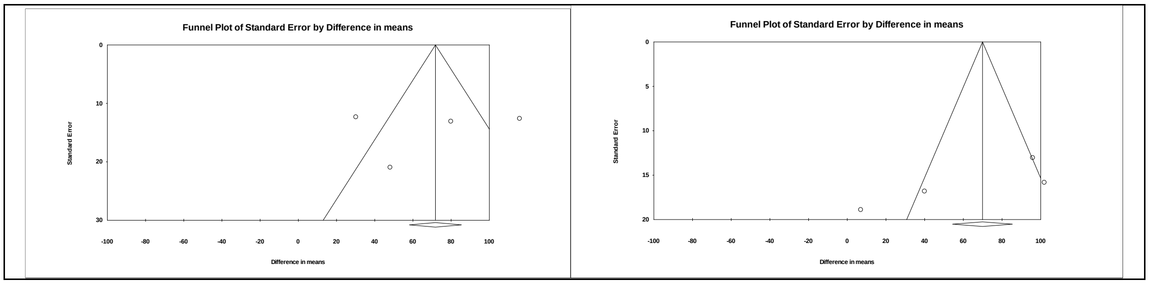

Figure S6: Funnel plot of publication bias on platelets for OZ (left) and ST (right) groups
